# Vortioxetine as an effective drug in the treatment of depression in adolescents with long QT index. A case report

**DOI:** 10.1192/j.eurpsy.2024.943

**Published:** 2024-08-27

**Authors:** P. Del Sol Calderon, A. Izquierdo de la Puente, R. Fernández, M. García Moreno

**Affiliations:** ^1^Psychiatry, Hospital Puerta de Hierro; ^2^Psychiatry, Hospital Universitario Infanta Cristina, Madrid, Spain

## Abstract

**Introduction:**

This is a 13-year-old female patient admitted to the psychiatric unit active suicidal ideation.

**Objectives:**

the objective is to show through a clinical case how vortioxetine can be safe in adolescents.

**Methods:**

Case report and literature review

**Results:**

She has a history of daily consumption of at least 2 units of cannabis per day. She presents high emotional distress secondary to academic failure, consuming the substance as a coping strategy. Due to prohibition and control by her parents, the patient stopped taking the substance, presenting severe depressive symptoms, self-injury and suicide ideation. For this reason she is admitted to the inpatient psychiatric unit. The electrocardiogram performed on admission shows a corrected QT index of 524. Exploring physical symptoms, she recognized episodes of syncope and palpitations. Coordination was made with cardiology, who performed an echocardiogram with normal results and began follow-up with them without prescribing medication. It was agreed not to use drugs that could prolong the QT index. Evaluating the clinical situation, it was decided to start treatment with Vortioxetine up to 10 mg. With this treatment there was no worsening of the electrocardiogram and the patient’s mood improved, anxiety and ideas of death were remitted

**Conclusions:**

This work aims to show how vortioxetine has been effective and safe at the cardiological level in the case of moderate-severe depression in an adolescent with prolonged QT index

**Disclosure of Interest:**

None Declared

